# Promises and challenges of applying large language models in the healthcare domain

**DOI:** 10.3389/fdgth.2026.1772274

**Published:** 2026-03-17

**Authors:** Qingyu Wang, Ziheng Gong, Zou Lai, Lina Bu, Fried-Michael Dahlweid, Hong Sun

**Affiliations:** 1 Provincial Key Laboratory of Multimodal Perceiving and Intelligent Systems, Jiaxing University, Jiaxing, China; 2 Sino-European Joint Lab for Health Information Processing and Applications, Jiaxing University, Jiaxing, China; 3 Dedalus Healthcare, Milan, Italy

**Keywords:** artificial intelligence, clinical decision support, large language models, model hallucinations, multimodal

## Abstract

Large language models are rapidly moving from theoretical concepts to active clinical pilots. Current approaches diverge between general-purpose models, which adapt to healthcare via prompt engineering, and domain-specific models, which prioritize deep alignment with medical knowledge graphs to ensure safety. Despite reported benefits in documentation efficiency and diagnostic reasoning, significant challenges remain regarding hallucination, privacy, and the validity of evaluation metrics. This Mini Review synthesizes current evidence, contrasts these two modeling paradigms, highlights key controversies, and maps out future development routes including retrieval-augmented generation and agentic architectures.

## 1 Introduction

Machine learning (ML)-based clinical risk prediction models have been widely developed for diverse medical applications (1). The advent of large language models (LLMs) boosted its application in healthcare domain in recent years, achieving close to human performances in benchmarks. Despite the fact that their real-world performance still falls short of experienced clinicians and requires rigorous oversight (2, 3), analyses of GPT-series models on medical licensure exams have validated their utility (4, 5). Consequently, there has been a surge in research applying LLMs to clinical documentation (6–8), health education (9), and diagnostic decision support (10–12).

Real-world applications are incorporating these models into clinical workflows, such as generating draft responses for EHR messaging (13); facilitating emergency handoffs (14), etc. Although these initiatives validate the technical feasibility of integration, they have also experienced problems such as load of the generated content, cognitive load, and legal liability (8, 15). Ultimately, both benchmark performance and pilot outcomes highlight enduring gaps in generalization, safety (issues from hallucinations), and ecological validity, especially given the current lack of rigorous governance (3, 11).

In light of these challenges, this mini-review synthesizes recent high-quality peer-reviewed literature to elucidate the evolutionary trajectory and paradigm shift from generalist foundation models to specialized medical LLMs (2, 3, 16). By integrating empirical evidence, we categorize current real-world applications into core domains, including diagnostic assistance, clinical documentation, and patient communication (2, 4, 6–9, 11–14, 17–33), and explore the current challenges, as well as how technological advancements are addressing these implementation hurdles.

**Figure 1:**
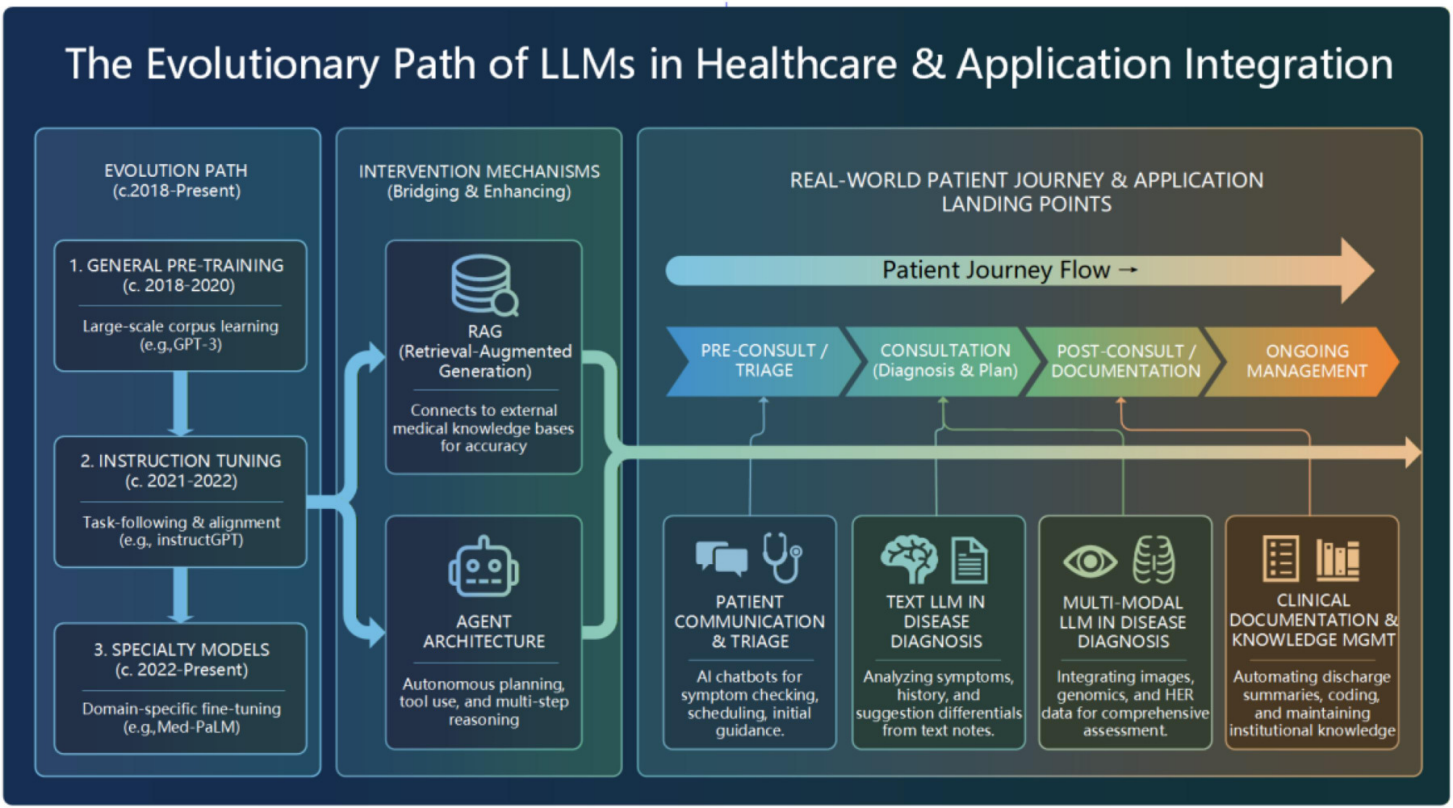
Systematic framework of Medical LLM Evolution and Application.

Figure 1 illustrates the trajectory from technical development to clinical implementation across three dimensions: The Evolution Path, tracing the shift from general pre-training to medical specialization; The Intervention Mechanisms, highlighting RAG and agentic architectures as bridges between capability and need; The Patient Journey Flow, mapping specific applications across triage, diagnosis (text/multi-modal), and documentation management.

## 2 Generalist and specialized LLM models in healthcare

Table 1 summarizes the technical landscape of generalist LLM models vs. specialized medical LLM models. While generalist models like GPT-4 have been widely tested for medical benchmarks (2, 34), the table highlights that open-source contenders such as DeepSeek are now approaching proprietary standards through architectural optimizations such as Mixture-of-Experts (MoE) (38, 40). Conversely, specialized models achieve superior knowledge fidelity and domain adaptation by prioritizing deep alignment on biomedical corpora (e.g., Med-PaLM 2) rather than sheer scale (3, 43, 49). Synthesizing these trends reveals a pivotal shift: the primary driver of clinical efficacy is moving from parameter size to alignment quality, pointing toward a synergistic future where the breadth of generalists and the domain depth of specialists function complementarily.

**Table 1:** Overview of representative large language models in healthcare.

Model	Dates Params	Access	Key technical & clinical characteristics	Ref.
I. Generalist Models: **General-purpose engines that apply broad world knowledge and reasoning to solve medical problems via prompt engineering or fine-tuning.**				
GPT-3.5	2022-11 Undisclosed	Closed	Early RLHF alignment; widely used benchmark for medical QA.	(1, 34–37)
Gemini	2023-12 Undisclosed	Closed	Long-context support; explores health coaching & decisions.	(38, 39)
GPT-4/4o	2023/2024 Undisclosed	Closed	Unified voice-image-text; SOTA performance on USMLE & radiology.	(2, 34, 37, 40)
DeepSeek-V3/R1	2024/2025 >500B	Open	MoE + Reasoning, high efficiency; strong in clinical deduction & psych eval.	(38, 40)
Qwen3	2025-04 0.6B–235B	Open	Separates memory/reasoning; 36T corpus + CoT/RL alignment.	(41, 42)
II. Specialized Models: **Healthcare domain specific systems deeply aligned with medical knowledge graphs and clinical data to ensure safety and terminological accuracy.**				
BioGPT	2022-11 1.5B	Open	PubMed Pre-training: Specialized in biomedical text mining & generation.	(43)
Med-PaLM 2	2023-05 340B	Closed	Aligned on MultiMedQA; approaches expert-level accuracy.	(2, 3, 37)
LLaVA-Med	2023-06 7B/13B	Open	Visual Alignment, fine-tuned on PMC-15M; focused on medical VQA.	(44, 45)
Med-PaLM M	2023-08 12B–562B	Closed	Modality-Agnostic, unified decoder for radiology, genomics, and text.	(46)
GatorTronGPT	2023-11 5B/20B	Semi-Open	EHR-Centric, trained from scratch on 82B clinical tokens; NLP focus.	(1, 47)
MedFound	2025-01 176B	Open	Bootstrapped CoT alignment for complex multi-specialty reasoning.	(16, 48)

## 3 Current landscape of applications

This section reviews the current landscape of Large Language Model (LLM) applications in healthcare. To provide a clear overview of the research frontier, we selected core literature based on impact, recency, and task representativeness, which are summarized in Table 2. We categorize these real-world applications into four primary domains:

**Table 2:** The applications of LLM in healthcare domain.

Category	Study	Model type	Specific task & key findings
I. Text-based Diagnosis	Rider et al. (50)	General LLM	Rare Disease: Six-LLM benchmark on primary immunodeficiencies; GPT-4o led with 96.2% diagnostic accuracy and strongest reasoning.
	Singhal et al. (2)	Specialized LLM (Med-PaLM 2)	Medical QA: Med-PaLM 2 reached 86.5% on MedQA, exceeding physician performance on multiple evaluation metrics.
	Liu et al. (48)	Specialized LLM (MedFound)	Generalist Medical Model: MedFound (176B) (clinical pretraining + CoT fine-tuning) outperformed baselines for both common and rare disease diagnosis.
II. Multimodal Diagnosis	Mao et al. (17)	Multimodal (GPT-4o)	Lung Nodule Follow-up: GPT-4o achieved strong longitudinal CT performance (Accuracy 0.88 malignancy; ICC 0.91 size measurement).
	Du et al. (51)	General LLM (Ensemble)	Cognitive Decline: GPT-4 + Llama 2 ensemble improved detection precision via complementary errors, outperforming single models.
III. Clinical Documentation	Hartman et al. (14)	General LLM (ChatGPT)	ER Handoff: LLM notes scored higher on automated metrics but slightly lower on expert safety, supporting human-in-the-loop use.
	Asgari et al. (52)	General LLM	Safety Framework: CREOLA framework validated on 13k sentences; low hallucinations (1.47%) and critical errors below human rates.
	Jin et al. (18)	LLM Agent	Subject Recruitment: TrialGPT delivered 87.3% expert-level trial matching and cut screening time by 42.6% (retrieval–ranking).
IV. Patient Communication	Daram et al. (9)	General LLM (ChatGPT)	Patient Education: Gynecology simplification increased conciseness but did not achieve the recommended 6th-grade reading level.
	Ayers et al. (19)	General LLM	Physician–Patient Empathy: AI chatbot replies preferred in 78.6% of cases, rated higher for quality and empathy than physicians.

### 3.1 Text-based LLMs in disease diagnosis

Text-based LLMs demonstrate mixed performance across diagnostic tasks. On one hand, generalist models like ChatGPT excel in standardized benchmarks, achieving 60%–80% accuracy on examinations such as the USMLE (4, 5, 53–55). On the other hand, in complex, real-world clinical scenarios, such as surgery recommendations or emergency decision-making, model performance often remains inferior to that of clinicians regarding diagnostic accuracy and guideline adherence (37, 56).

Furthermore, current models remain constrained by hallucinations (37, 53, 57). Consensus suggests that the primary value of text-based LLMs currently lies in information synthesis and alleviating administrative burdens; their deployment as independent diagnostic agents remain hindered by the need for robust reliability verification frameworks (37, 52).

### 3.2 Multimodal LLMs in disease diagnosis

In contrast to text-only models, multimodal LLMs demonstrate the potential for cross-modal integration and diagnostic assistance. Zhang et al. achieved near-radiologist performance in zero-shot diagnosis by aligning imaging with clinical reports via knowledge graphs (24). In oncology, vision-language models show promise for early lung cancer screening and prognosis prediction (30, 34).

However, significant limitations persist. First, data and interpretability gaps remain: models often rely on single-center data, and cross-modal alignment mechanisms lack the clinical explainability, which is required to establish physician trust (1, 2, 10). Second, validation is insufficient: there is a lack of systematic comparisons with established deep learning networks (10, 37). Future work is required to strengthen data standardization, interpretability, and multi-center validation (10, 52, 58).

### 3.3 Clinical documentation and knowledge management

LLMs demonstrate substantial potential in documentation generation and knowledge extraction. Tools have been verified to significantly reduce charting time and EHR interaction duration through real-time dialogue transcription (1, 8, 26, 28). Additionally, LLMs excel in generating high-quality clinical notes, extracting clinical trial eligibility criteria, and simplifying complex patient documents (6, 7, 9, 43).

Nevertheless, their utility remains a subject of debate. First, studies indicate that efficiency gains are heterogeneous across specialties, and existing evidence often lacks rigorous control groups or transparency (1, 13). Second, models may lack domain specific details and remain highly sensitive to prompting strategies, necessitating human-in-the-loop review (2, 32). Therefore, while promising, LLMs are best positioned as assistive tools rather than independent agents, awaiting improvements in cross-disease generalization and error control (52, 53).

### 3.4 Patient communication and triage

In the domain of patient communication and triage, LLMs are increasingly deployed to assist in emergency triage, generate educational materials, and facilitate online consulting, thereby enhancing accessibility and reducing communication burdens. Ayers et al. utilized real-world patient queries from Reddit to compare physician and AI responses, finding that evaluators preferred AI responses in the majority of cases due to higher information quality and empathetic tone (19). This highlights a distinct advantage of LLMs in drafting empathetic medical communications. Similarly, Zaretsky et al. demonstrated that GPT-4 can effectively translate discharge summaries into patient-friendly language, significantly improving readability and health literacy comprehension (33).

## 4 Challenges

Based on the application scenarios outlined above, the deployment of Large Language Models (LLMs) in healthcare continues to face five common and critical challenges:

### 4.1 Reliability and hallucination control

Studies indicate that LLMs are prone to hallucinations, generating factually incorrect information with high confidence, particularly in complex or information-scarce scenarios. This poses a direct safety threat to diagnostic and therapeutic recommendations (20, 21, 57). Even models that perform adequately on standardized benchmarks often demonstrate instability when handling heterogeneous real-world cases or executing multi-step clinical reasoning (2, 21, 37, 53). This highlights the need for safety-specific evaluations on realistic cases, and human-in-the-loop deployment for high-stakes decisions.

### 4.2 Lack of validation framework

Current evaluations are predominantly confined to offline technical metrics, lacking a hierarchical validation framework that bridges general capabilities, task-specific performance, and real-world clinical outcomes (22, 52). Existing studies are frequently limited by small sample sizes, the absence of proper control groups, or a lack of clear clinical endpoints, making the evidence insufficient to support the adoption of models as formal medical interventions (2, 10, 37).

### 4.3 Data bias and completeness

Mainstream models are largely trained on open databases, common disease, resulting in insufficient coverage of rare diseases, and specific underrepresented populations. This necessitates supplementation with specialized data (1, 10, 43). Furthermore, the opacity of training data sources risks exacerbating existing medical and societal biases (2, 59).

### 4.4 Ethical and governance dilemmas

Privacy preservation and algorithmic fairness present significant challenges in API-based deployment. As highlighted by Ong et al, developers and healthcare professionals must share responsibility and justice throughout the model's entire lifecycle (60).

### 4.5 Ecosystem and path selection

Close-source commercial models face inherent limitations regarding auditing and local deployment. Conversely, an open and transparent ecosystem is more conducive to long-term safety regulation and oversight (61). The medical community should actively participate in data governance and the definition of clinical requirements.

## 5 Summary

In summary, transforming LLMs to robust clinical infrastructure requires systematic efforts centered on data quality, uncertainty management, hierarchical validation, and ethical governance. These measures are essential to ensuring that models deliver value within a strict framework of human-AI collaboration and evidence-based medicine (22, 37, 60–63).

## 6 Future perspectives

While Large Language Models (LLMs) hold immense potential for disease risk prediction and broader healthcare scenarios, they entail significant application risks. Future research and practice must collaborate towards exploring knowledge enhancement, decision synergy, and ecosystem construction.

First, Retrieval-Augmented Generation (RAG) offers a viable pathway to mitigate model hallucinations. By integrating clinical guidelines and literature as external knowledge sources, RAG grounds predictions in traceable evidence rather than relying solely on parametric memory. Future efforts should prioritize building specialized retrieval databases centered on Electronic Health Records (EHRs), follow-up data, and multimodal inputs. This will enable the simultaneous presentation of risk assessments and their evidentiary sources, thereby enhancing the reliability of clinical conclusions (64–66).

Second, **Agentic Architectures** are promising in reshaping clinical decision workflows. Unlike standalone LLMs that only generate text, **agentic systems are semi-autonomous**: they can plan multi-step actions and **invoke external resources** to produce evidence-linked outputs. In this paradigm, an LLM can act as a coordinating hub, extracting structured information, calling task-specific models for precise computation, and synthesizing results at a macro level, while maintaining appropriate human oversight in high-stakes settings (23, 67).

Third, Semantic Web and Knowledge Graph technologies can serve as the foundation for deep clinical understanding. Ontology-based semantic modeling can organize multidimensional elements into computable knowledge networks. This not only provides precise retrieval boundaries for RAG and agent systems but also offers structured support for causal modeling in risk prediction.

Finally, the Open-Source Ecosystem, exemplified by models like DeepSeek, and Qwen, presents new opportunities for privatized deployment. Unlike closed-source counterparts, open-source LLMs support fine-tuning and deployment within local environments, effectively mitigating data sovereignty and privacy risks. Furthermore, the availability of multiple parameter scales allows healthcare institutions to maintain an optimal balance between performance and cost based on their specific needs. Although the recent release of models like DeepSeek implies a lag in published validation studies, the rapid evolution of the open-source ecosystem is expected to significantly accelerate the practical adoption of LLMs in healthcare.

## 7 Conclusion

This review systematically delineated the technological evolutionary path of large language models from general pre-training to medical specialization and compared their efficacy across diagnostic, documentation, and communication scenarios. Our analysis suggests that the future paradigm of medical AI will not be a binary choice between generalist and specialized models, but rather a convergence: generalist models will leverage their robust reasoning to handle complex, unstructured clinical contexts, while specialized medical models will serve as the expert kernel providing precise knowledge and safety boundaries.

Although severe challenges remain regarding data transparency, hallucination control, and reliable validation systems, the advancement of RAG, agentic architectures, and the open-source ecosystem is gradually transforming LLMs from standalone conversational tools into embedded clinical infrastructure. Future research must focus on establishing rigorous validation frameworks and, under the premise of ethical compliance, driving the technology to systemic synergy.
